# Supplementary material for: Role of Sox3 in Estradiol-Induced Sex Reversal in Pelodiscus sinensis
Source: Int J Mol Sci. 2023 Dec 23;25(1):248. doi: 10.3390/ijms25010248 (PMC10779075; doi:10.3390/ijms25010248)
Supplement: Supplementary file 1 [file ijms-25-00248-s001.zip › ijms-2741769-supplementary.pdf]

Supplementary Figure S1

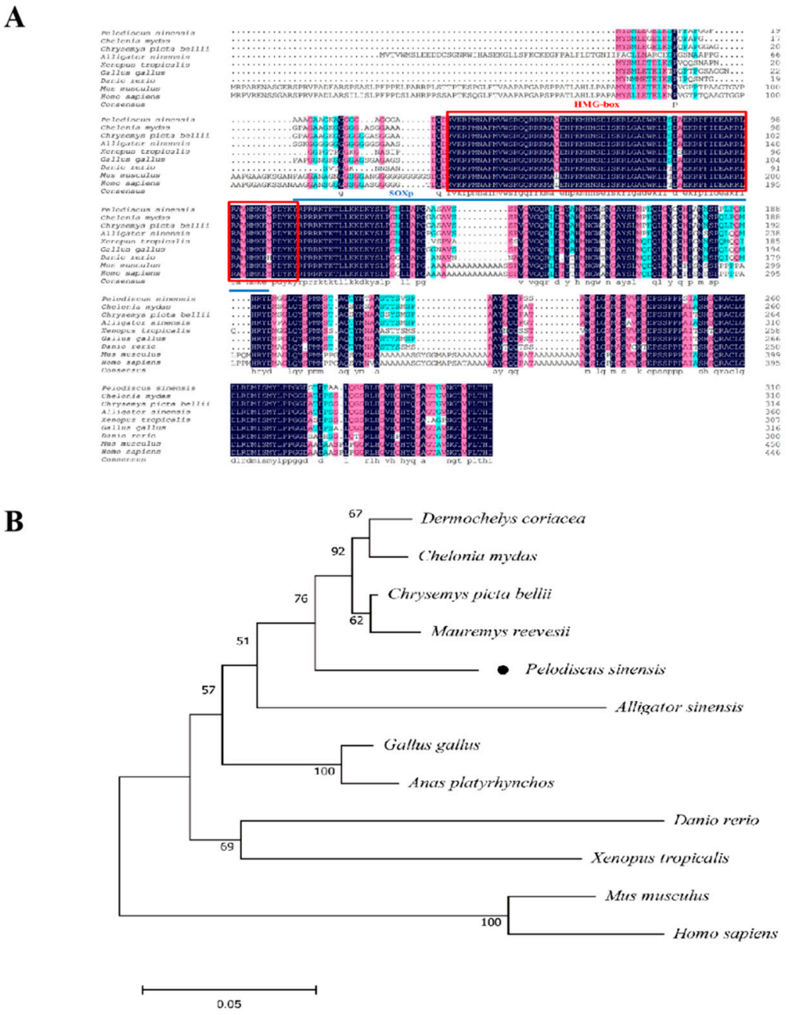

Supplementary Figure 1. The amino acid sequence alignment and phylogenetic tree of *P. sinensis* Sox3 gene and other species.(A)Amino acid sequence alignment of *P. sinensis* Sox3 gene with other species. (B) Phylogenetic tree of *P. sinensis* Sox3 gene and other species. The red box represents the HMG box DNA-binding domain; the blue line represents the SOXp motif. "●" means P. sinensis. D. coriacea (XM_038417687.1); C. mydas (XM_037908813.1); C. picta bellii (XM_005294617.2); M. reevesii (XM_039488955.1); A. sinensis (XM_006031059.1); G. gallus (NM_204195.1); A. platyrhynchos (XM_027465289.2); D. rerio (NM_001001811.2); X. tropicalis (NM_001007501.1); M. musculus (NM_009237.2); H. sapiens (NM_005634.3).

Supplementary Figure S2

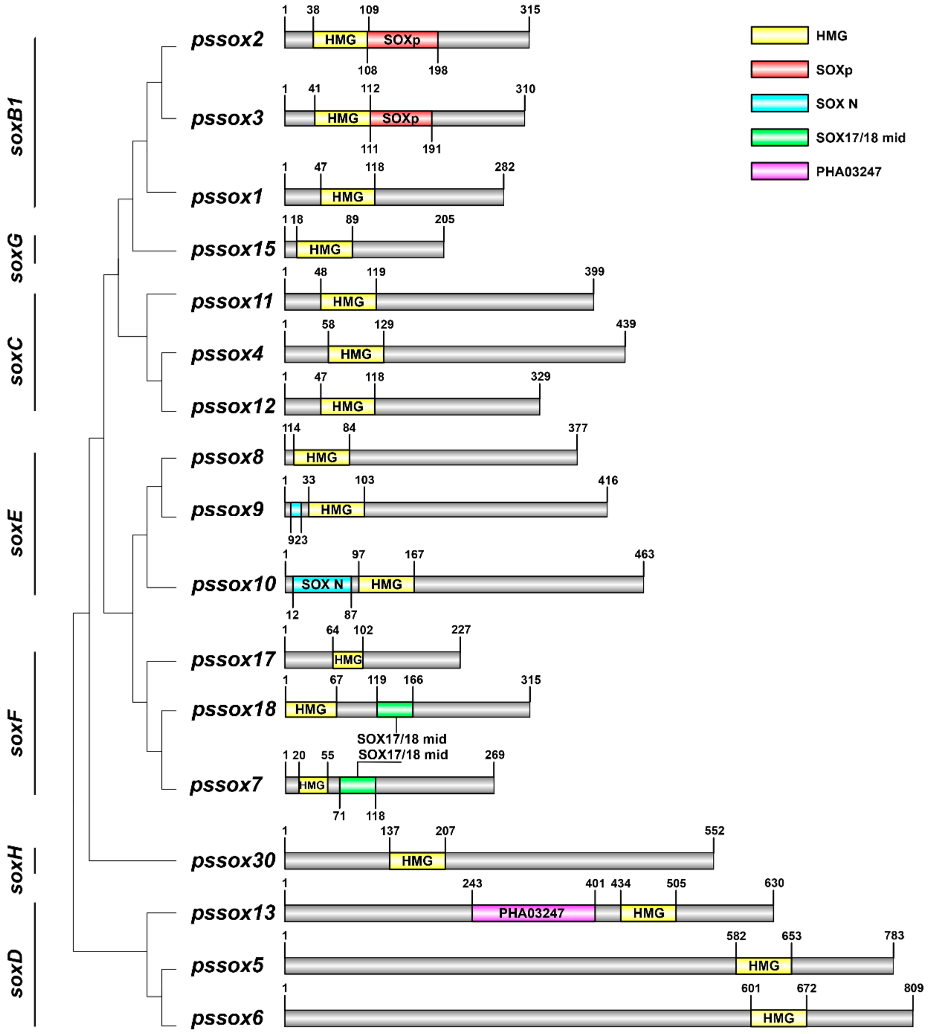

Supplementary Figure S2. The functional structure of *Sox* family.

Supplementary Figure S3

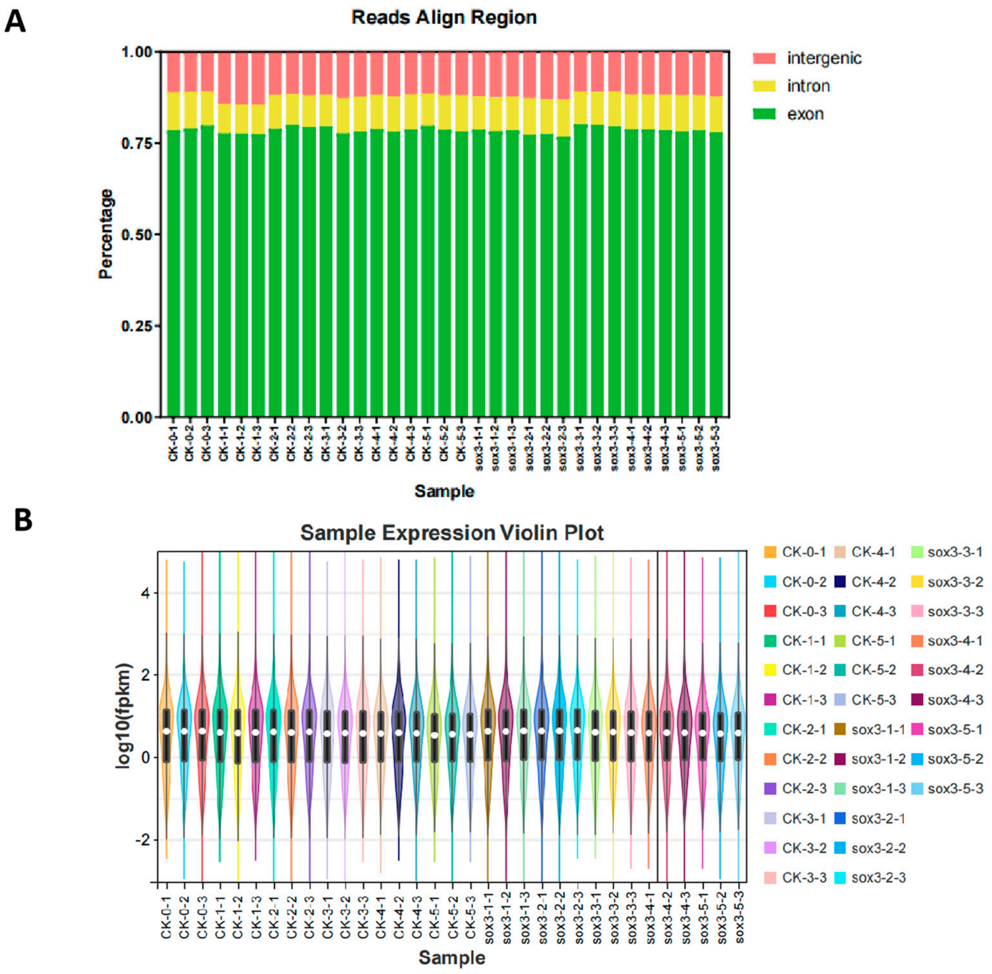

Supplementary Figure 3. The reads align region and the expression violin plot of all samples.(A) The reads align region of all samples.(B) The expression violin plot of all samples.

Supplementary Figure S4

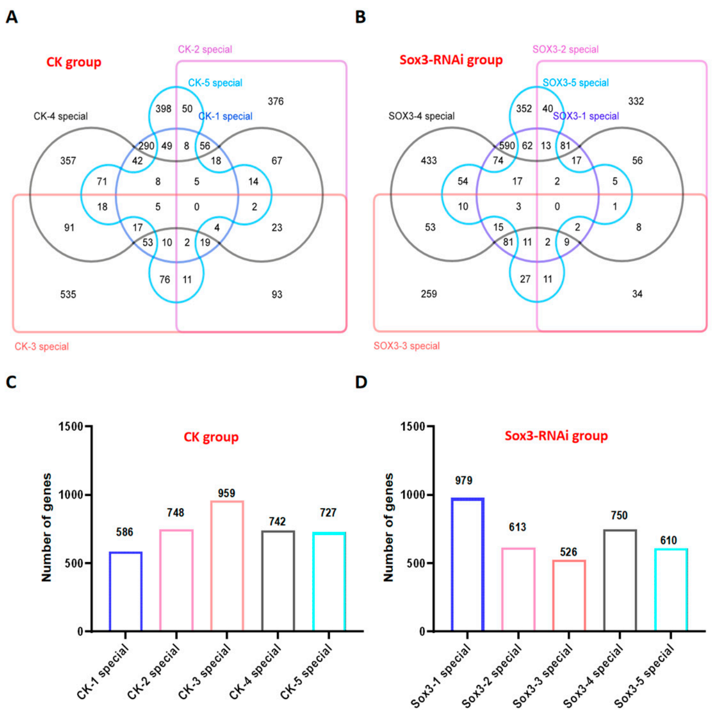

Supplementary Figure 4. The comparative analysis of the specially expressed genes in CK group or *Sox3*-RNAi group. (A) The Wayne analysis of specially expressed genes in CK group at different developmental stages. (B) The Wayne analysis of specially expressed genes in *Sox3*-RNAi group at different developmental stages. (C) The number of specially expressed genes in CK group at different developmental stages. (D) specially expressed genes in *Sox3*-RNAi group at different developmental stages.

Supplementary Tab.S1 PCR Primer sequences used in this experiment.

**Primer Name**	**Primer Sequence（5'-3'）**	**Application**
Sox3-F1<br>*Sox3*-R1	GCAAGATGGCCCAGGAGAAC<br>TCCATTCCACCTCTACACTCAT	PCR
Sox3-GSP5-1	TCTTCAGCAGGGTCTTGGTCTTCC	5′ RACE
Sox3-GSP3-1	AGTACAGCCCCATGATGTCCACG	3′ RACE
Sox3-GSP3-2	TTCCTGGTTTCTGATAGGTTGGCG	
UPM	CTAATACGACTCACTATAGGGCAAGCAGTGGTATCAACGCAGAGT	RACE
UPM short	CTAATACGACTCACTATAGGGC	
Sox3-qF	ACACCTACGCGCACATGAA	*Sox3* RT-qPCR
Sox3-qR	CATGTCGTAGCGGTGCATCT	
Sox9-qF	TACGACTACACCGACCACCA	*Sox9* RT-qPCR
Sox9-qR	GTAGTGTCTGCAATGGGCGT	
Wnt4-qF	GAGGTGATGGACTCGGTGCG	*Wnt4* RT-qPCR
Wnt4-qR	CCCGTTCTTGAGGTCGTGGTC	
Dkk4-qF	TTTGCTGTGCTCGCCATTTC	*Dkk4* RT-qPCR
Dkk4-qR	AAGCGAGACTGTTGTGGCAT	
Nog-qF	ATCTAATCGAGCACCCGGAC	*Nog* RT-qPCR
Nog-qR	CTCCAGCCCTTTGATTTCGC	
Mmp11-qF	ACACGGATTTTGACGCTGTT	*Mmp11* RT-qPCR
Mmp11-qR	CCAGATATTGCCTCGGGAGT	
Ptchd3-qF	TCAGCCTAATCGCTCTCGC	*Ptchd3* RT-qPCR
Ptchd3-qR	GCTCCATACAGCAAAACCACA	
Cbx2-qF	GGACTGTGAACCTGGCAAAG	*Cbx2* RT-qPCR
Cbx2-qR	GTGAGCTTTGGGGCTCTTTC	
Sppl3-qF	GCTGTTGTGGACGTTTCACT	*Sppl3* RT-qPCR
Sppl3-qR	TAACCCCGAAAGCAGCAAAC	
Msi1-qF	CGATCGACCCAAAAGTAGCG	*Msi1* RT-qPCR
Msi1-qR	CATCGCATCATCCACCTTCC	
Krt14-qF	TCTCGTGGACGTCAAGACAC	*Krt14* RT-qPCR
Krt14-qR	CCTCCTCAAGAACCGTGTGG	
Ccm1-qF	ATGAGGGTCTGCAGCGAATC	*Ccm1* RT-qPCR
Ccm1-qR	CTCTGTCTGCCCAGGTTCAA	
Ranbp1-qF	AGGAACCATTCGCCTCCTTA	*Ranbp1* RT-qPCR
Ranbp1-qR	AAAGCGGATTGCCAGAAGTT	
Npffr2-qF	TCTCGCTATCGCCATCATGT	*Npffr2* RT-qPCR
Npffr2-qR	GAGTGAAAGGGGAGCCAGAT	
Mapk10-qF	GTGCATCATGGGCGAAATGA	*Mapk10* RT-qPCR
Mapk10-qR	TGGGAAAGTGAGACCAGCATAC	
Gapdh-qF	AGAACATCATTCCAGCATCCA	Internal reference
Gapdh-qR	CTTCATCACCTTCTTAATGTCGTC	
SOX3-567<br>*SOX3-765*<br>*SOX3-986*	GCTGAAGAAGGACAAATACTC<br>GCAGATGCACCGCTACGACAT<br>GGGACCTGCGGGATATGATCA	Internal reference

Supplementary Tab.S2 The specially expressed genes existed in at least four periods in CK group.

Embryonic development stage	Symbol	Description
S15. S16. S17. S18.	*LOC102462307*	Uncharacterized gene
	Slc10A1	Sodium/bile acid cotransporter
S14. S16. S17. S18.	*Mrc1*	Macrophage mannose receptor 1
	Cacng8	Calcium voltage-gated channel auxiliary subunit gamma 8
	Gje1	Gap junction protein epsilon 1
	LOC112545474	Uncharacterized gene
	LOC112545639	Uncharacterized gene
S14. S15. S17. S18.	*MSTRG.41164*	Uncharacterized gene
	Map7	Microtubule associated protein 7
	Bpifb4	BPI fold-containing family B member 4
	Brd8	Bromodomain containing 8
S14. S15. S16. S18.	*P2Ry6*	Pyrimidinergic receptor P2Y6
	MSTRG.2377	Uncharacterized gene
	Spaca3	Sperm acrosome associated 3
	Sun3	Sad1 and UNC84 domain containing 3
S14. S15. S16. S17.	*LOC106732602*	Uncharacterized gene
	LOC112545130	Uncharacterized gene
	LOC106732525	Uncharacterized gene

Supplementary Tab.S3 The specially expressed genes existed in at least four periods in *Sox3*-RNAi group.

Embryonic development stage	Symbol	Description
S15. S16. S17. S18.	*LOC106732672*	Phospholipase A2 inhibitor and Ly6/PLAUR domain-containing protein-like
S14. S16. S17. S18	*Fam47E*	Protein FAM47E
	Gfer	Growth factor, augmenter of liver regeneration
	Srrm4	Serine/arginine repetitive matrix 4
S14. S15. S17. S18.	*Pcdha8*	Protocadherin alpha 8
	LOC106731389	Uncharacterized gene
S14. S15. S16. S18.	*Tuba1B*	Tubulin alpha-1B chain
	Znf24	Zinc finger protein 24
S14. S15. S16. S17.	*LOC102448701*	Uncharacterized gene
	Cd48	CD48 molecule

Supplementary Tab.S4 Top 15 downregulated expressed genes between CK group and *Sox3*-RNAi group.

**Upregulated genes**	**Description**
Msi1	musashi RNA binding protein 1
Krt14	keratin 14
Ccm1	Ccm1p
Ranbp1	RAN binding protein 1
Npffr2	Neuropeptide FF receptor 2
Mapk10	Mitogen-activated protein kinase 10
Sost	Sclerostin
Cyp27b1	Cytochrome P450 family 27 subfamily B member 1
Pla2g4c	Phospholipase A2 group IVC
Ache	Acetylcholinesterase
Csf3r	Colony-stimulating factor 3 receptor
Ccr5	C-C motif chemokine receptor 5
Nox1	NADPH oxidase 1
Rac2	Rac family small GTPase 2
Alox5	Arachidonate 5-lipoxygenase

Supplementary Tab.S5 Top 15 upregulated expressed genes between CK group and *Sox3*-RNAi group

**Downregulated genes**	**Description**
Dkk4	*Dickkopf WNT signaling pathway inhibitor 4*
Nog	*Noggin*
Mmp11	*Matrix metallopeptidase 11*
Ptchd3	*Patched domain containing 3*
Cbx2	*Chromobox 2*
Sppl3	*Signal peptide peptidase-like 3*
Cyp24a1	*Cytochrome P450 family 24 subfamily A member 1*
Fgf19	*Fibroblast growth factor 19*
Efna2	*Ephrin-A2*
P2rx3	*Purinergic receptor P2X 3*
Bax	*BCL2 associated X, apoptosis regulator*
Mapk10	*Mitogen-activated protein kinase 10*
Gnao1	*G protein subunit alpha o1*
Nlrp1b	*NLR family, pyrin domain containing 1B*
Gria4	*Glutamate ionotropic receptor AMPA type subunit 4*
